# Supplementary material for: A new reproductive mode in anurans: Natural history of Bokermannohyla astartea (Anura: Hylidae) with the description of its tadpole and vocal repertoire
Source: PLoS One. 2021 Feb 17;16(2):e0246401. doi: 10.1371/journal.pone.0246401 (PMC7888631; doi:10.1371/journal.pone.0246401)
Supplement: S1 Appendix — (DOCX) [file pone.0246401.s001.docx]

**A new reproductive mode in anurans: natural history of *Bokermannohyla astartea* (Anura: Hylidae) with the description of its tadpole and vocal repertoire**

Leo R. Malagoli, Tiago L. Pezzuti, Davi L. Bang, Julián Faivovich, Mariana L. Lyra, João G. R. Giovanelli, Paulo C. A. Garcia, Ricardo J. Sawaya, Célio F. B. Haddad

*Plos One*

**S1 Appendix.** **Specimens of tadpoles, spawn and adults examined.** Abbreviations: CFBH = Coleção de Anfíbios “Célio F. B. Haddad” (CFBH), Departamento de Biodiversidade, Instituto de Biociências, Universidade Estadual Paulista; Museu de Zoologia da Universidade de São Paulo (MZUSP); Museu Nacional da Universidade Federal do Rio de Janeiro (MNRJ); ZUEC-AMP = Coleção de Anfíbios, Museu de Zoologia, Instituto de Biologia, Universidade Estadual de Campinas; UFMG = Coleção Herpetológica da Universidade Federal de Minas Gerais (UFMG).

**Tadpoles and spawn**

*Bokermannohyla astartea*. Brazil, State of São Paulo, municipality of São Paulo, Parque Estadual da Serra do Mar - Núcleo Curucutu CFBH 38047 (spawning with 105 eggs), CFBH 38055 (lot of five tadpoles in stages 33−36), CFBH 42648 (lot of seven tadpoles in stages 25−28), CFBH 42649 (lot of three tadpoles in stages 31−36), CFBH 42650 (lot of three tadpoles in stages 27−36), CFBH 42651 (lot of three tadpoles in stages 31−37), CFBH 42652 (lot of three tadpoles in stages 28−35), CFBH 42653 (lot of one tadpole in stage 36), CFBH 42654 (lot of three tadpoles in stage 36), CFBH 42655 (lot of two tadpoles in stages 35−36), CFBH 42656 (lot of two tadpoles in stages 26−27), CFBH 42657 (lot containing one egg and two tadpoles in stage 25), CFBH 42658 (lot of ten tadpoles in stages 23−26), CFBH 42659 (lot of 12 tadpoles in stages 25−26), CFBH 42660 (lot containing spawning with 74 eggs and 12 tadpoles in stages 23−24); municipality of Itanhaém, Parque Estadual da Serra do Mar - Núcleo Curucutu CFBH 42661 (lot of two tadpoles in stages 30−33); municipality of Salesópolis, Estação Biológica de Boracéia CFBH 39948 (lot containing spawning with 172 eggs, and one tadpole in stage 25).

*Bokermannohyla* gr. *circumdata*. Brazil, State of São Paulo, municipality of Itanhaém, Parque Estadual da Serra do Mar - Núcleo Curucutu CFBH 45165 (lot of one tadpole in stage 43), CFBH 45166 (lot of one tadpole in stage 44), CFBH 45167 (lot of one tadpole in stage 45), CFBH 45168 (lot of one tadpole in stage 45), CFBH 45169 (lot of six tadpoles in stages 25−35), CFBH 45170 (lot of three tadpoles in stages 25−35), CFBH 45172 (lot of one tadpole in stage 36), CFBH 45174 (lot of two tadpoles in stages 26−32), CFBH 45175 (lot of one tadpole in stage 36), CFBH 45176 (lot of two tadpoles in stages 32−36), CFBH 45177 (lot of one tadpole in stage 36), CFBH 45179 (lot of one tadpole in stage 35), CFBH 45180 (lot of three tadpoles in stages 29−36), CFBH 45181 (lot of two tadpoles in stages 28−30), CFBH 45182 (lot of one tadpole in stage 31), CFBH 45184 (lot of 27 tadpoles in stages 26−36), CFBH 45185 (lot of 22 tadpoles in stages 27−39).

*Aplastodiscus* aff. *albosignatus*. Brazil, State of São Paulo, municipality of Itanhaém, Parque Estadual da Serra do Mar - Núcleo Curucutu CFBH 45171 (lot of one tadpole in stage 36), CFBH 45178 (lot of one tadpole in stage 30), CFBH 45183 (lot of one tadpole in stage 34).

**Adults**

*Bokermannohyla astartea*. Brazil, State of São Paulo, municipality of Santo André, Reserva Biológica de Paranapiacaba MZUSP 74196 (holotype, male), MZUSP 74221−22 (paratypes, males); Paranapiacaba MNRJ 4052 (paratype, male), ZUEC-AMP 6401; municipality of Salesópolis, Estação Biológica de Boracéia MZUSP 137455, MZUSP 2820−21 (males), MZUSP 3856 (female), MZUSP 60192−93 (males), MZUSP 2542, MZUSP 34585−87, MZUSP 34592 (males), MNRJ 18850−53 (males), CFBH 39957−60 (males); municipality of São Miguel Arcanjo, Parque Estadual Carlos Botelho MZUSP 136541(male), CFBH 38447 (male); Tapiraí CFBH 15570−72 (males); municipality of São Paulo, Parque Estadual da Serra do Mar - Núcleo Curucutu CFBH 11167−69, CFBH 11184 (males), CFBH 36191 (female), CFBH 36354−55, CFBH 38044 (males), CFBH 38045 (female), CFBH 38046, CFBH 42053 (males), CFBH 42054 (female); municipality of Itanhaém, Parque Estadual da Serra do Mar - Núcleo Curucutu CFBH 42055−57 (males), UFMG 20150 (male).
