# Supplementary material for: A new reproductive mode in anurans: Natural history of Bokermannohyla astartea (Anura: Hylidae) with the description of its tadpole and vocal repertoire
Source: PLoS One. 2021 Feb 17;16(2):e0246401. doi: 10.1371/journal.pone.0246401 (PMC7888631; doi:10.1371/journal.pone.0246401)
Supplement: S1 Table — All recordings were obtained at Núcleo Curucutu, Parque Estadual da Serra do Mar, state of São Paulo, southeastern Brazil. (DOCX) [file pone.0246401.s002.docx]

**A new reproductive mode in anurans: natural history of *Bokermannohyla astartea* (Anura: Hylidae) with the description of its tadpole and vocal repertoire**

Leo R. Malagoli, Tiago L. Pezzuti, Davi L. Bang, Julián Faivovich, Mariana L. Lyra, João G. R. Giovanelli, Paulo C. A. Garcia, Ricardo J. Sawaya, Célio F. B. Haddad

*Plos One*

| **Number FNJV** | **Type of call** | **Municipality** | **Breeding**  **site (BS)** | **Recording date (mm/dd/yyyy)** | **Recording time** | **Voucher specimen** | **Air/water temperature (°C)** |
| --- | --- | --- | --- | --- | --- | --- | --- |
| 45382 | Advertisement | São Paulo | BS1 | 09/23/2014 | 2013 h | –– | 14.9 |
| 45380 | Advertisement | São Paulo | BS1 | 10/13/2014 | 1907 h | –– | 22.3 |
| 45385 | Advertisement | São Paulo | BS1 | 10/14/2014 | 1915 h | –– | 20.8 |
| 45386 | Advertisement | São Paulo | BS1 | 10/14/2014 | 1942 h | CFBH 38044 | 20.6/20.9 |
| 45387 | Advertisement | São Paulo | BS1 | 10/14/2014 | 1955 h | –– | 20.6 |
| 45388 | Amplectant | São Paulo | BS1 | 10/14/2014 | 2350 h | CFBH 38044 | 18.7 |
| 45389 | Advertisement | Itanhaém | BS2 | 10/15/2014 | 1843 h | –– | 17.8 |
| 45391 | Advertisement | Itanhaém | BS2 | 10/15/2014 | 2050 h | –– | 17 |
| 45390 | Advertisement | São Paulo | BS1 | 03/11/2015 | 1930 h | CFBH 42053 | 20.1 |
| 45392 | Advertisement | Itanhaém | BS2 | 03/12/2015 | 2056 h | CFBH 42055 | 20/20.8 |
| 45381 | Advertisement | Itanhaém | BS2 | 12/28/2016 | 1930 h | UFMG 20150 | 22.3 |
| 45383 | Advertisement | Itanhaém | BS2 | 12/28/2016 | 2006 h | CFBH 42057 | 22 |
| 45384 | Presumable territorial | Itanhaém | BS1 | 12/28/2016 | 2015 h | CFBH 42057 | 22 |

**S1 Table. Metadata of sound files and type of calls analyzed of *Bokermannohyla astartea*.** All recordings were obtained at Núcleo Curucutu, Parque Estadual da Serra do Mar, state of São Paulo, southeastern Brazil. Abbreviations: FNJV = Fonoteca Neotropical Jacques Vielliard, Museu de Zoologia, Instituto de Biologia, Universidade Estadual de Campinas; CFBH = Coleção de Anfíbios “Célio F. B. Haddad” (CFBH), Departamento de Biodiversidade, Instituto de Biociências, Universidade Estadual Paulista, Rio Claro, SP; UFMG = Coleção Herpetológica da Universidade Federal de Minas Gerais (UFMG).
