# Supplementary material for: A new reproductive mode in anurans: Natural history of Bokermannohyla astartea (Anura: Hylidae) with the description of its tadpole and vocal repertoire
Source: PLoS One. 2021 Feb 17;16(2):e0246401. doi: 10.1371/journal.pone.0246401 (PMC7888631; doi:10.1371/journal.pone.0246401)
Supplement: S2 Table — Tissue sample ID, GenBank accession numbers (sequences generated in this study), molecular identification, identity, most similar sequence and respective voucher, lot number of voucher specimen, and collection breeding site and respective environment. (DOCX) [file pone.0246401.s003.docx]

**A new reproductive mode in anurans: natural history of *Bokermannohyla astartea* (Anura: Hylidae) with the description of its tadpole and vocal repertoire**

Leo R. Malagoli, Tiago L. Pezzuti, Davi L. Bang, Julián Faivovich, Mariana L. Lyra, João G. R. Giovanelli, Paulo C. A. Garcia, Ricardo J. Sawaya, Célio F. B. Haddad

*Plos One*

**S2 Table. Tadpole identification with metadata information.** Tissue sample ID, GenBank accession numbers (sequences generated in this study), molecular identification, identity, most similar sequence and respective voucher, lot number of voucher specimen, and collection breeding site and respective environment. Abbreviations: CFBH = Coleção de Anfíbios “Célio F. B. Haddad” (CFBH), Departamento de Biodiversidade, Instituto de Biociências, Universidade Estadual Paulista, Rio Claro, SP; CFBHT = “Célio F. B. Haddad” Tissue collection; USNM = National Museum of Natural History.

| **Tissue sample ID** | **GenBank** | **Molecular identification** | **Identity (%)** | **Most similar sequence; respective voucher** | **Lot number (CFBH)** | **Collection breeding site (BS); environment** |
| --- | --- | --- | --- | --- | --- | --- |
| CFBHT 23052 | MH201231 | *Bokermannohyla astartea* | 99.65 | MH201228 *Bokermannohyla astartea*; CFBH 38045 | 38055 | BS1; bromeliads |
| CFBHT 23053 | MH201232 | *Bokermannohyla* gr. *circumdata* | 99.27 | AY549328 *Bokermannohyla circumdata*; CFBH 3621 | 45165 | BS2; stream |
|  |  |  | 99.27 | AY549338 *Bokermannohyla hylax*; USNM 303036 |  |  |
| CFBHT 23054 | MH201233 | *Bokermannohyla* gr. *circumdata* | 100 | KU495160 *Bokermannohyla circumdata*; CFBHT 14585 | 45166 | BS2; stream |
|  |  |  | 99.15 | AY549338 *Bokermannohyla hylax*; USNM 303036 |  |  |
| CFBHT 23055 | MH201234 | *Bokermannohyla* gr. *circumdata* | 98.9 | AY549338 *Bokermannohyla* hylax; USNM 303036 | 45167 | BS2; stream |
|  |  |  | 98.53 | AY549328 *Bokermannohyla circumdata*; CFBH 3621 |  |  |
| CFBHT 23056 | MH201235 | *Bokermannohyla* gr. *circumdata* | 100 | KU495160 *Bokermannohyla circumdata*; CFBHT 14585 | 45168 | BS2; stream |
|  |  |  | 99.22 | AY549338 *Bokermannohyla hylax*; USNM 303036 |  |  |
| CFBHT 23057 | MH201236 | *Bokermannohyla astartea* | 100 | MH201228 *Bokermannohyla astartea*; CFBH 38045 | 42649 | BS1; stream |
| CFBHT 23058 | MH201237 | *Bokermannohyla astartea* | 99.83 | MH201229 *Bokermannohyla astartea*; CFBH 38447 | 42649 | BS1; stream |
| CFBHT 23059 | MH201238 | *Bokermannohyla astartea* | 99.83 | MH201229 *Bokermannohyla astartea*; CFBH 38447 | 42650 | BS1; stream |
| CFBHT 23060 | MH201239 | *Bokermannohyla astartea* | 99.65 | MH201229 *Bokermannohyla astartea*; CFBH 38447 | 42652 | BS1; stream |
| CFBHT 23061 | MH201240 | *Bokermannohyla astartea* | 100 | MH201228 *Bokermannohyla astartea*; CFBH 38045 | 42652 | BS1; stream |
| CFBHT 23062 | MH201241 | *Bokermannohyla astartea* | 100 | MH201228 *Bokermannohyla astartea*; CFBH 38045 | 42653 | BS1; stream |
| CFBHT 23063 | MH201242 | *Bokermannohyla astartea* | 100 | MH201228 *Bokermannohyla astartea*; CFBH 38045 | 42654 | BS1; bromeliads |
| CFBHT 23064 | MH201243 | *Bokermannohyla astartea* | 99.83 | MH201229 *Bokermannohyla astartea*; CFBH 38447 | 42655 | BS1; stream |
| CFBHT 23065 | MH201244 | *Bokermannohyla astartea* | 99.83 | MH201229 *Bokermannohyla astartea*; CFBH 38447 | 42656 | BS1; stream |
| CFBHT 23066 | MH201245 | *Bokermannohyla* gr. *circumdata* | 99.29 | AY549328 *Bokermannohyla circumdata*; CFBH 3621 | 45169 | BS2; stream below bromeliads |
|  |  |  | 99.11 | AY549338 *Bokermannohyla hylax*; USNM 303036 |  |  |
| CFBHT 23067 | MH201246 | *Bokermannohyla* gr. *circumdata* | 99.11 | AY549328 *Bokermannohyla circumdata*; CFBH 3621 | 45169 | BS2; stream below bromeliads |
|  |  |  | 98.93 | AY549338 *Bokermannohyla hylax*; USNM 303036 |  |  |
| CFBHT 23068 | MH201247 | *Bokermannohyla* gr. *circumdata* | 99.27 | AY549328 *Bokermannohyla circumdata*; CFBH 3621 | 45170 | BS2; stream below bromeliads |
|  |  |  | 99.27 | AY549338 *Bokermannohyla hylax*; USNM 303036 |  |  |
| CFBHT 23069 | MH201248 | *Bokermannohyla* gr. *circumdata* | 99.29 | AY549328 *Bokermannohyla circumdata*; CFBH 3621 | 45170 | BS2; stream below bromeliads |
|  |  |  | 99.11 | AY549338 *Bokermannohyla hylax*; USNM 303036 |  |  |
| CFBHT 23070 | MH201249 | *Bokermannohyla astartea* | 100 | MH201228 *Bokermannohyla astartea*; CFBH 38045 | 42658 | BS1; bromeliads |
| CFBHT 23071 | MH201250 | *Bokermannohyla astartea* | 99.65 | MH201228 *Bokermannohyla astartea*; CFBH 38045 | 42658 | BS1; bromeliads |
| CFBHT 23072 | MH201251 | *Aplastodiscus* aff. *albosignatus* | 99.82 | KU184004 *Aplastodiscus* sp. 5; CFBH 11183 | 45171 | BS2; stream |
| CFBHT 23073 | MH201252 | *Bokermannohyla* gr. *circumdata* | 99.27 | AY549328 *Bokermannohyla circumdata*; CFBH 3621 | 45172 | BS2; stream |
|  |  |  | 99.27 | AY549338 *Bokermannohyla hylax*; USNM 303036 |  |  |
| CFBHT 23074 | MH201253 | *Bokermannohyla* gr. *circumdata* | 99.03 | AY549338 *Bokermannohyla hylax*; USNM 303036 | 45173 | BS2; stream |
|  |  |  | 99.03 | KU495154 *Bokermannohyla circumdata*; CFBHT 01051 |  |  |
| CFBHT 23075 | MH201254 | *Bokermannohyla* gr. *circumdata* | 100 | KU495160 *Bokermannohyla circumdata*; CFBHT 14585 | 45174 | BS2; stream below bromeliads |
|  |  |  | 99.22 | AY549338 *Bokermannohyla hylax*; USNM 303036 |  |  |
| CFBHT 23076 | MH201255 | *Bokermannohyla* gr. *circumdata* | 100 | KU495160 *Bokermannohyla circumdata*; CFBHT 14585 | 45175 | BS2; stream below bromeliads |
|  |  |  | 99.22 | AY549338 *Bokermannohyla hylax*; USNM 303036 |  |  |
| CFBHT 23077 | MH201256 | *Bokermannohyla astartea* | 99.81 | MH201228 *Bokermannohyla astartea*; CFBH 38045 | 42661 | BS2; stream below bromeliads |
| CFBHT 23078 | MH201257 | *Bokermannohyla astartea* | 98.32 | MH201228 *Bokermannohyla astartea*; CFBH 38045 | 42661 | BS2; stream below bromeliads |
| CFBHT 23079 | MH201258 | *Bokermannohyla* gr. *circumdata* | 100 | KU495160 *Bokermannohyla circumdata*; CFBHT 14585 | 45176 | BS2; stream |
| CFBHT 23080 | MH201259 | *Bokermannohyla* gr. *circumdata* | 99.1 | KU495154 *Bokermannohyla circumdata*; CFBHT 01051 | 45177 | BS2; stream |
|  |  |  | 98.81 | AY549338 *Bokermannohyla hylax*; USNM 303036 |  |  |
| CFBHT 23081 | MH201260 | *Aplastodiscus* aff. *albosignatus* | 99.61 | KU184004 *Aplastodiscus* sp.; CFBH 11183 | 45178 | BS2; stream |
| CFBHT 23082 | MH201261 | *Bokermannohyla* gr. *circumdata* | 97.36 | AY549338 *Bokermannohyla hylax*; USNM 303036 | 45179 | BS2; stream |
|  |  |  | 97.36 | KU495154 *Bokermannohyla circumdata*; CFBHT 01051 |  |  |
| CFBHT 23083 | MH201262 | *Bokermannohyla* gr. *circumdata* | 98.82 | AY549338 *Bokermannohyla hylax*; USNM 303036 | 45180 | BS2; stream |
|  |  |  | 98.82 | KU495154 *Bokermannohyla circumdata*; CFBHT 01051 |  |  |
| CFBHT 23084 | MH201263 | *Bokermannohyla* gr. *circumdata* | 99.41 | KU495154 *Bokermannohyla circumdata*; CFBHT 01051 | 45180 | BS2; stream |
|  |  |  | 99.02 | AY549338 *Bokermannohyla hylax*; USNM 303036 |  |  |
| CFBHT 23085 | MH201264 | *Bokermannohyla* gr. *circumdata* | 100 | KU495160 *Bokermannohyla circumdata*; CFBHT 14585 | 45181 | BS2; stream |
|  |  |  | 99.22 | AY549338 *Bokermannohyla hylax*; USNM 303036 |  |  |
| CFBHT 23086 | MH201266 | *Bokermannohyla* gr. *circumdata* | 98.82 | AY549338 *Bokermannohyla hylax*; USNM 303036 | 45182 | BS2; stream |
|  |  |  | 98.82 | KU495154 *Bokermannohyla circumdata*; CFBHT 01051 |  |  |
| CFBHT 23087 | MH201265 | *Aplastodiscus* aff. *albosignatus* | 100 | KU184004 *Aplastodiscus* sp. 5; CFBH 11183 | 45183 | BS2; stream |
| CFBHT 23088 | MT509986 | *Bokermannohyla* gr. *circumdata* | 99.27 | AY549328 *Bokermannohyla circumdata*; CFBH 3621 | 45184 | BS2; stream below bromeliads |
|  |  |  | 99.27 | AY549338 *Bokermannohyla hylax*; USNM 303036 |  |  |
| CFBHT 23089 | MT509987 | *Bokermannohyla* gr. *circumdata* | 99.28 | AY549328 *Bokermannohyla circumdata*; CFBH 3621 | 45185 | BS2; stream below bromeliads |
|  |  |  | 99.10 | AY549338 *Bokermannohyla hylax*; USNM 303036 |  |  |
