# Supplementary material for: A new reproductive mode in anurans: Natural history of Bokermannohyla astartea (Anura: Hylidae) with the description of its tadpole and vocal repertoire
Source: PLoS One. 2021 Feb 17;16(2):e0246401. doi: 10.1371/journal.pone.0246401 (PMC7888631; doi:10.1371/journal.pone.0246401)
Supplement: S3 Table — (DOCX) [file pone.0246401.s004.docx]

**A new reproductive mode in anurans: natural history of *Bokermannohyla astartea* (Anura: Hylidae) with the description of its tadpole and vocal repertoire**

Leo R. Malagoli, Tiago L. Pezzuti, Davi L. Bang, Julián Faivovich, Mariana L. Lyra, João G. R. Giovanelli, Paulo C. A. Garcia, Ricardo J. Sawaya, Célio F. B. Haddad

*Plos One*

**S3 Table. Sequential behavioral features of the five observed courtships of *Bokermannohyla astartea*.**

|  | **Courtship 1** | **Courtship 2** | **Courtship 3** | **Courtship 4** | **Courtship 5** |
| --- | --- | --- | --- | --- | --- |
| **Date** | Nov. 26 2013 | Oct. 14 2014 | Oct. 14 2014 | Oct. 31 2014 | Mar. 11 2015 |
| **Air temperature** | 17.9 °C | 18.8 °C | 18.2 °C | 18.7 °C | 19.6 °C |
| **Start time; duration of each sequence** | 2025 h; about 25 min. | 2055 h; about 75 min. | 2310 h; about 50 min. | 2045 h; about 60 min. | 1935 h; about  100 min. |
| **Presence of satellite male** | no | yes | yes | no | no |
| **Leaf-tank inspection by female** | yes | no | yes | yes | yes |
| **Amplexus** | no | no | yes | yes | yes |
| **Clutch partition between leaf-tank bromeliads** | no | no | yes | yes | no |
| **Additional observations** | no | no | no | no | Male rubs its gular region on the female's head, while in amplexus |
