# Supplementary figures and images for: A new reproductive mode in anurans: Natural history of Bokermannohyla astartea (Anura: Hylidae) with the description of its tadpole and vocal repertoire
Source: PLoS One. 2021 Feb 17;16(2):e0246401. doi: 10.1371/journal.pone.0246401 (PMC7888631; doi:10.1371/journal.pone.0246401)

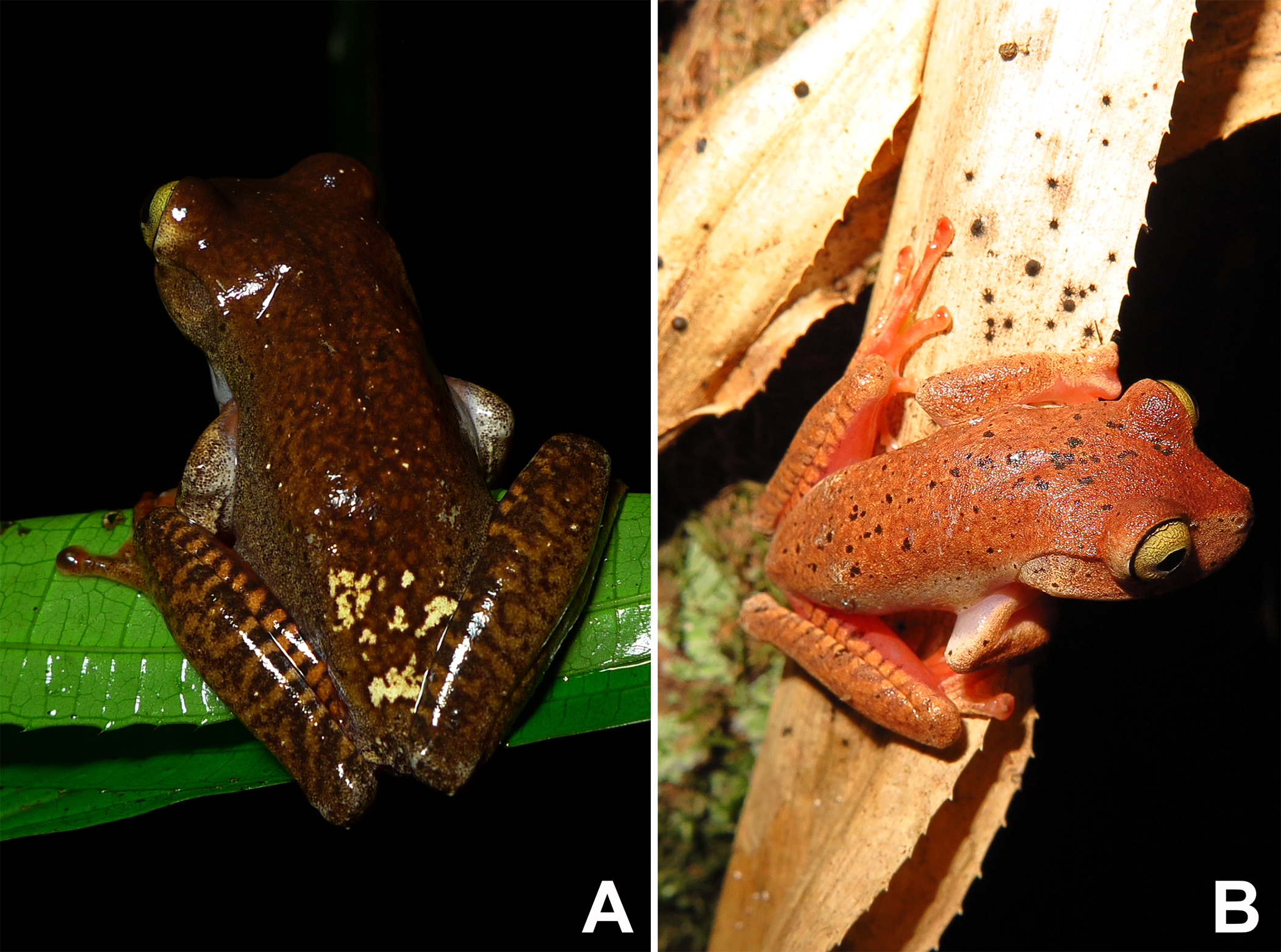

Supplement: S1 Fig — (A) Male with white blotches on the posterior portion of the dorsum and (B) male with black spots scattered on the dorsum. (TIF) [file pone.0246401.s005.tif]

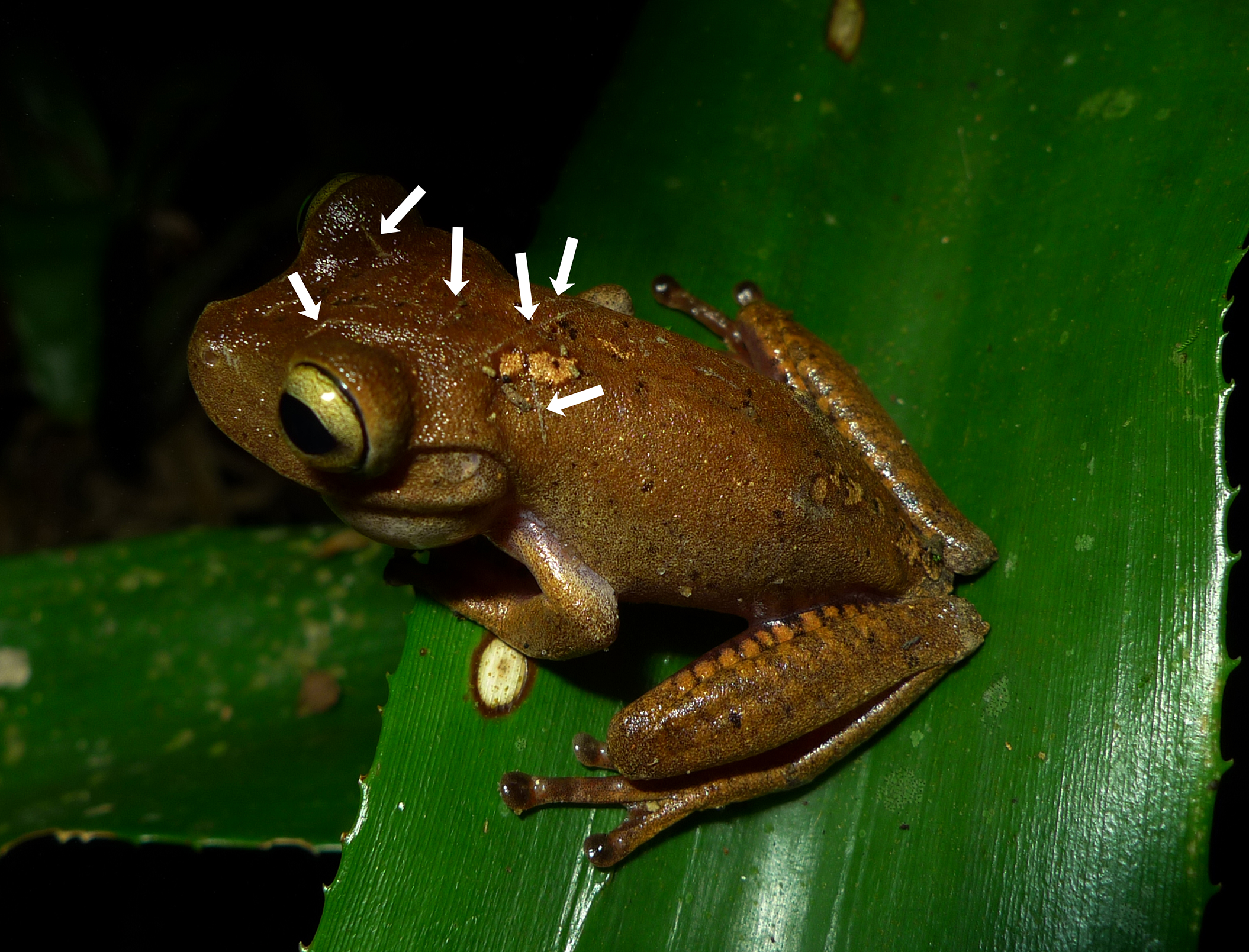

Supplement: S2 Fig — White arrows indicate some of the scars. (TIF) [file pone.0246401.s006.tif]
