# Supplementary material for: A new reproductive mode in anurans: Natural history of Bokermannohyla astartea (Anura: Hylidae) with the description of its tadpole and vocal repertoire
Source: PLoS One. 2021 Feb 17;16(2):e0246401. doi: 10.1371/journal.pone.0246401 (PMC7888631; doi:10.1371/journal.pone.0246401)
Supplement: Attachment — Submitted filename: Malagoli_et_al_Response_to_Reviewers.docx [file pone.0246401.s010.docx]

**Response to Reviewers for the MS Malagoli *et al*. A new reproductive mode in anurans: natural history of *Bokermannohyla astartea* (Anura: Hylidae) with the description of its tadpole and vocal repertoire**, which is under review in *PlosOne* (**PONE-D-20-19802**).

**Editor:**

1.Thank you for submitting your manuscript to PLOS ONE. After careful consideration, we feel that it has merit but does not fully meet PLOS ONE’s publication criteria as it currently stands. Therefore, we invite you to submit a revised version of the manuscript that addresses the points raised during the review process.
There were two referees who both find the results interesting and suggest that they should be published. Also, on the one hand, there are mainly minor comments regarding the manuscript's structure, language etc.

However, on the other hand, one referee made an important point (and therefore even ranked the paper 'reject'), and I share this point. There is a lot of natural history information on a particular species and the focus of the paper is not really strong. That is, the paper as it is would perhaps work well in a herp journal but not so well in a more general science journal. The one major point of your findings, the new reproductive mode, is certainly something special and something beyond a natural history study. But this is not well emphasized given the amount of other information. The referee suggests to split the information into various papers, which I think is a good idea.

Along with this, the introdcution and the discussion remain a bit too general in the paper as it is. These critics are NOT a reason to reject your paper according to the publication criteria of PLoS ONE. As a result I ranked your paper 'minor revision' and leave the decision with you to perhaps shorten the manuscript for PLoS ONE with a stronger focus on the reproductive mode novelty.

**Answer:** We carefully reviewed each question and suggestions, trying to include them in the revised manuscript. We have reduced the size of the manuscript removing the sections of ecological modeling, distribution and conservation, and also the section about defensive behavior, reducing as well the number of figures from 11 to 7. We have also restructured the Introduction and the Discussion making it more focused on the new reproductive mode, to make it more attractive to broader viewers.

**Reviewer #1**

1. This study brings novel and important information on the biology and distribution of a frog species, including the description of a new reproductive mode for anurans. The manuscript is a beautiful natural history study and is well written. Although I recognize the importance of natural history studies to enhance our knowledge on the ecology and evolution of organisms, I have to say that the manuscript suffers from the lack of focus, which makes it extremely long. Additionally, the work is centered in the species biology, which makes it less attractive to a broader audience.
As the study presents diverse aspects of the species biology, I think the authors should consider separating the content of the manuscript in different more focused manuscripts (e.g. reproductive mode and breeding biology; description of tadpole and call types; geographic distribution and conservation). I would like to emphasize that my suggestion is not based solely on the length of the work, but because shorter and more focused studies are more likely to be read and to reach greater impact in the scientific community. Below, I provide some suggestions that might be useful to broaden the focus of the manuscript.

**Answer:** Thank you for the all the valuable suggestions. We have suppressed the sections concerning to defensive behaviors, geographic distribution, and conservation of the species, and this has reduced the text size and consequently the number of figures, from 11 to 7. Therefore, we tried to put focus in the new reproductive mode, which inherently englobes the species reproductive biology. Although the suggestion to separate the content of our work is appreciated, we deemed that data on tadpoles and calls of the species to be of crucial importance to supplement our description of the new reproductive mode. Moreover, we believe that the description of the only bromeligenous tadpole of the Cophomantini tribe, can add more value to the manuscript, considering that the description of the new reproductive mode involves the change in the tadpole's development habitat, implying differences in the stage of development and size of the tadpoles in each environment (*i*.*e*., bromeliads and in streams/ponds). In this sense, despite the manuscript is now more focused in the species reproductive biology, we restructured Introduction and Discussion sections in an attempt to reach more broaden audience.

2. The introduction is very restricted to the taxon studied. It is possible to modify the framework to make the study more interesting to a broader readership. For instance, the great diversity of reproductive modes in frogs could be introduced in the light of the evolution of terrestrial reproduction in vertebrates and the factors leading to terrestriality. Furthermore, other aspects of the study are not presented in the introduction. The importance of acoustic communication and diversity of acoustic repertoires in many animal groups, including anurans, could be mentioned in the introduction. Similarly, as the manuscript uses predictive models to infer geographic distribution and discuss the conservation status of the species, these subjects should be presented in the introduction (e.g. the situation of amphibian conservation in the world and in Brazil, main threats to the group, the current conservation status of the species and the importance of predicting geographic distribution to support conservation policies).

**Answer:** We agree with the suggestions made. We have reformulated the introduction to make it more comprehensive and attractive to broader readership. We present some points related to the evolution of terrestrial reproduction in vertebrates and the factors leading to terrestriality, including anurans. Additionally, we have included information about the importance of describing tadpoles and knowledge about the natural history of larvae in the context of reproductive modes. Likewise, as suggested, we pointed out on the importance of communication and acoustic repertoire for different animal groups, including anurans. With the intention of making our work more focused in the new reproductive mode, we removed the Geographical distribution and conservation section, and respective results and discussion of the manuscript.

3. Similarly to the introduction, the discussion section presents a superficial discussion because of the great variety of subjects in the manuscript. For instance, in the first paragraph of the discussion, if water bodies are available, I was wondering about the factors that could have led to the reproduction in bromeliads in this species (e.g. to avoid competition with other species in aquatic sites, avoid predation, avoid competitor males or sneakers?). There are recent papers discussing the evolution of terrestrial reproduction in frogs that could be mentioned. Furthermore, in the last years, new reproductive modes have been described for anurans and currently we have more than 40 reproductive modes (mentioned in the introduction), many of them described for species from tropical forests outside the Neotropics. Factors related to the evolution of this striking diversity of reproductive modes among tropical frogs could also be discussed.

**Answer:** We agree with the suggestions made. At the end of the discussion section, we included a “Remarks” section, formerly named “Concluding remarks”. In this section we include all the suggestions made by the Reviewer, addressing the issues in a more broadly sense, trying to discuss the traits of the species' newly reproductive biology in relation with the factors that could have driven the evolution and diversification of terrestrial/arboreal reproductive modes in anurans.

**Reviewer #2**

1. The study by Malagoli et al. is a very nice piece of amphibian natural history, well written and richly and beautifully illustrated. It describes a new reproductive mode in anurans, courtship, defensive behaviors, spawning, and tadpoles of *Bokermannohyla astartea*. They also update the description of its vocal repertoire and provide updated information on its geographic distribution and conservation status. There is some criticism surrounding the description of new reproductive modes in amphibians because very subtle differences in reproductive biology are sometimes used to propose a ´new reproductive mode´. The reproductive mode of *B*. *astartea* seems, however, indeed distinctive. Of course, there is an important element missing… that is, how do tadpoles end up in surface waterbodies. Maybe the authors could be more explicit about whether they have invested the time and effort needed to find tadpoles above stage 26 in bromeliads, or tadpoles below stage 26 in streams. Descriptions of behavior, tadpoles and calls (based on my limited experience with bioacoustics) are all very thorough. Again, the pictures and, to be honest, all graphical elements, are excellent. I only have minor comments that could be addressed to improve the paper.

**Answer:** We really appreciate all the suggestions and corrections. We agree with the observation that the way the tadpoles end up in the waterbodies from the bromeliads is still unknown. However, through the information we present about the characteristics of its breeding environment, its reproductive biology, and the description of its tadpole, we highlight that the most likely scenario is that the tadpole jump or is transported after the overflow of leaf-tanks of bromeliads to the main waterbody, considering that the streams and rivulets are always located very close to the sets of bromeliads. In addition, we also better emphasize differences in the developmental stages of tadpoles when they are in bromeliads and when they are in streams in the discussion section (in subsection Reproductive mode, spawning, and tadpoles in bromeliads and in streams), also pointing that tadpoles beyond stage 26 were never found within bromeliads. We emphasized in the methods, in the “spawn, egg, and tadpole” section, that the investment in the search for tadpoles occurred in both bromeliads and streams.

2. Line 118 - Seventy-one-days exclusively or predominantly devoted to observing *B*. *astartea*? Or 71 days in which several tasks were conducted in the field including observing B. astarea?

**Answer:** It was 71 days exclusively to observe *B*. *astartea*. We better detailed this information in the text.

3. Line 119 – Observations were done in only two breeding sites? Is this species of difficult access in other localities within the range of its distribution?

**Answer:** Yes, the observations were done focally in two breeding sites in our work. This species is not easily found within the range of its distribution, occurring in specific environments that are not always accessible and not easily to find in the field.

4. Lines 133 – 145 – Figure titles for figures 1 and 2 are the same. I believe you could change them to better indicate what each figure shows. Alternatively, you could rearrange those two panels into a figure that shows the breeding sites and its differences (temporary vs permanent streams? BS1 vs BS2?) and another showing the position of calling activity on the BSs.

**Answer:** We agree with the change of text in the titles of figures 1 and 2. We changed the titles to better indicate what each figure shows as suggested, but we chose to keep the figures in the same arrangement because we consider that they are relevant to illustrate both the types of habitats used by the species as well as the relationship with the new reproductive mode described.

5. Line 147 - What is a ´all-occurrence sampling method´?

**Answer:** This method records all types of behaviors performed by a species during a given time period of observation. We included this information in the text to make it clearer.

6. Lines 164-165 - Liver and muscle samples were preserved… What for?
**Answer:** We added the sentence: “for DNA extraction protocols”.

7. Lines 167 – 170 – How many specimens in each category (resident males, satellite males, females)?

**Answer:** We inform how many specimens were measured in each category in the respective sections of results, both in the “Temporal breeding pattern and habitat use” section and in the “Male site fidelity, satellite behavior, and territoriality” section.

8. Lines 191, 626, 687 - Presumed? Presumably?

**Answer:** We agree that the correct word is presumably and we corrected it throughout the text.

9. Lines 228 a 244 – It is not absolutely necessary, but It would be helpful for readers to have the measurements of the external morphology indicated in a figure like Figure 6.

**Answer:** We included the measurements of the total length of the tadpoles in the captions of Figure 5 (previously referred to as Figure 6). For the figure 6 (previously referred to as Figure 7), which presented details of some external characters of the tadpole, we do not include the measurements of each external character in the figure legend, because it is uncommon to show these measurements in the captions of this type of figure. To make the reader go (if desired) to the table where all the measurements are present, we include the following sentence in the caption: “The measurements of external characters are presented in Table 2”.

10. Lines 309 – 311 – Did they use 75% of the data to train the algorithms and 25% to validate it? Please, explain in a bit more detail.

**Answer:** We removed the Geographical distribution and conservation section and respective results and discussion of the manuscript.

11. Lines 339 – 341 – When you show the number of individuals (n), also provide percentages so we can immediately know how rare or common the behavior was in your sample size. Like you did in “Males vocalizing outside bromeliads (6.7%, n = 6)”.
**Answer:** We agree. We included the percentages that were missing in the respective observations.

12. Line 345 –“39.7 ± 1.3 mm (36.8–42.7 mm, n = 40)”. What are these intervals inside the parenthesis? Minimum and maximum values? Confidence intervals?

**Answer:** In this case the interval within the parenthesis are the minimum and maximum values of SVL. At the end of the methods section, we included an explanatory sentence in relation to the values presented throughout the text: “Descriptive statistics are henceforth shown as mean ± standard deviation, (minimum–maximum (when applicable), and number of individuals or specific records).”.

13. Line 363 – “(n = 14, % of total males observed?)”

**Answer:** We agree. We added the sentence: “15,7% of total males observed in study”.

14. Line 366-369. I don’t think you have to indicate the full study locality in the figure title. Just “Males of *Bokermannohyla astartea* recaptured in the same sets of bromeliads in the two breeding sites sampled (BS1 and BS2)” would be enough.

**Answer:** We agree. We removed the sentence: “from Núcleo Curucutu, Parque Estadual da Serra do Mar, in the municipalities of São Paulo and Itanhaém, state of São Paulo, Brazil, respectively”.

15. Line 399. The male does not spawn. I assume you mean ´after egg laying was completed´ or something like that.

**Answer:** We agree. We replaced the term spawning by “egg laying was completed”.

16. Lines 427-428. Is the satellite male observing or being observed? I believe you meant “no satellite males observing” here.

**Answer:** We apologize. What we meant in this sentence is that in this courtship observation, we did not observe any satellite male. We excluded the word “being” from the sentence.

17. Line 443. ´ Spawns were always found in bromeliad leaf-tanks´ - as opposed to? All your sampling effort was invested into bromeliads. Would you have found spawns in a surface waterbody?

**Answer:** Opposed to finding spawns in streams, which were also part of the sampling effort. We complemented the sentence including that the spawns of *B*. *astartea* were not found in the streams. We also included and detailed in the methods section the information about the sampling efforts that we invested in both bromeliads leaf-tanks and streams.

18. Line 449 – “(139–194)” minimum and maximum values?

**Answer:** Yes, minimum and maximum values of mature oocytes. We changed the placement of this range of values to directly after the values of mean and standard deviation, which we think now will be clearer that it refers to minimum and maximum values of mature oocytes.

19. Line 535 – 538. I cannot fully see four posterior tooth rows or the gap in the first tooth row in Figure 7A. This is not necessarily a problem and I know those pictures are hard to obtain. Still, do you have another picture that clearly shows them?

**Answer:** We appreciate the concern with the improvement of the figure. Unfortunately, this was the only individual photographed, and we could not open the oral disc a bit more without damaging it. However, we think that although it is not completely open, it is possible to count the posterior rows (P4, P3, P2, from the lateral regions, P1 in the medial region) and the gap in P1 can be visualized discretely in the medial region enlarging the figure. We could graphically indicate these structures, but the design was not good, polluting the figure a lot. Thus, we believe that the figure is sufficient to show that there are 4 rows and a gap at P1.

20. Lines 647 – Again, you do not need to repeat the locality in the title of the table.

**Answer:** Done, we removed the sentence: “from Núcleo Curucutu, Parque Estadual da Serra do Mar, in the municipalities of São Paulo and Itanhaém, southeastern Brazil” from the table caption and all other figures or tables captions that we had included this sentence in the first version.

21. Line 752 – Remove comma in “Even, the predicted occurrence”

**Answer:** We removed the Geographical distribution and conservation section and respective results and discussion of the manuscript.
