# Supplementary material for: A new reproductive mode in anurans: Natural history of Bokermannohyla astartea (Anura: Hylidae) with the description of its tadpole and vocal repertoire
Source: PLoS One. 2021 Feb 17;16(2):e0246401. doi: 10.1371/journal.pone.0246401 (PMC7888631; doi:10.1371/journal.pone.0246401)
Supplement: Attachment — Submitted filename: Malagoli_et_al_Response_to_Reviewers.docx [file pone.0246401.s011.docx]

**Response to Reviewers for the MS Malagoli *et al*. A new reproductive mode in anurans: natural history of *Bokermannohyla astartea* (Anura: Hylidae) with the description of its tadpole and vocal repertoire**, which is under review in *PlosOne* (**PONE-D-20-19802R1**).

**Editor:**

1. Thank you for submitting your manuscript to PLOS ONE. After careful consideration, we feel that it has merit but does not fully meet PLOS ONE’s publication criteria as it currently stands. Therefore, we invite you to submit a revised version of the manuscript that addresses the points raised during the review process.

There are some few issues left that need attention. The one referee and myself were very satisfied with your review.

**Answer:** Thank you for all suggestions and corrections. We seek to address most of the issues raised, which we present below.

**Reviewer #1**

1. This is a beautiful natural history paper, describing a new reproductive mode for a Neotropical anuran species and other reproductive traits. I really appreciated reading this revised version. The authors did a great job improving the introduction and the discussion. I think that the manuscript can be accepted after minor revision, mostly regarding text corrections and adjustments. Although I tried to correct some grammar errors, I thnk that the manuscript still needs an English revision. My specific suggestions/corrections and comments follow below.

**Answer:** We are very grateful for your review that improved the quality of our manuscript. We carefully reviewed each question and suggestions, trying to include them in the revised version of manuscript. For issues that have not been fully addressed, we have justified and discussed them thoroughly. We also made a new English revision.

**Abstract**

2. Line 47: Include “the” before “novel reproductive mode”.

**Answer:** Done.

**Introduction**

3. Lines 54-59: In the paper by Silva et al. (2020), authors suggest that the reproductive modes have influenced fecundity and sexual dimorphism in anurans, not the opposite as stated in the introduction. Thus, I suggest rewriting the sentence excluding this part, as follows:
“However, recent studies indicate that sexual selection by male-male competition [5] and parental care [6] also played an important role in the evolution of terrestrial reproductive modes in anuran amphibians, indicating that multiple factors can contribute towards terrestriality.” You can also join the first two paragraphs.

**Answer:** Done. As suggested, we rewrote the sentence and join the first two paragraphs.

4. Line 61: replace “tetrapods”

**Answer:** Done.

5. Line 76: replace “…is the diversity in tadpole morphology”

**Answer:** Done.

6. Line 78: substitute “upon” for “on”

**Answer:** Done.

7. Line 85: substitute “to” for “in”

**Answer:** Done.

8. Line 86: delete “categorized as”
**Answer:** Done.

9. Lines 88-90: rewrite as follows: “…and territorial calls may have unique traits and play determinant roles within the social context and reproductive biology of anurans”
**Answer:** Done. We rewrited the sentence as suggested.

10. Line 95: include “a genus of” before gladiator treefrog

**Answer:** Done.

11. Line 101: include “the” before reproductive biology

**Answer:** Done.

12. Line 107: include “the” before Serra do Mar

**Answer:** Done.

13. Line 116: include “the” before reproduction

**Answer:** Done.

**Materials and methods**

14. Line 132: it seems that montane is repeated
**Answer:** Done. We excluded the repeated word.

15. Line 180: when referring to live animals, it is better to use “individuals, males, females, etc”. Specimen is mainly used for dead animals deposited in collections. Check throughout the text.
**Answer:** Done. We corrected the use of the word “specimen” throughout the text. Then, as suggested, we used “exemplars, individuals, males, etc” to refer to live animals.

16. Lines 191-192: If you used these tissue samples from adults for tadpole identification, it would be nice to clearly state that “These samples were used for tadpole molecular identification (see details below).”
**Answer:** Done. We included the suggested sentence.

17. Line 245: replace “are given”

**Answer:** Done.

18. Line 286: It would be better to write “…traits were measured according to Köhler et al…”

**Answer:** Done.

**Results**

19. Line 315: exclude “and” before Vriesea and insert a comma
**Answer:** Done.

20. Lines 312-314: You have beautiful pictures, thus you could refer to Fig. 2 when describing calling sites.
**Answer:** Done.

21. Lines 323-326: The first paragraph of the results describes temporal breeding pattern and habitat use. Thus, results on male and female SVL and sexual dimorphism fit better in the second paragraph.

**Answer:** Done. We included this part of the results in a second paragraph.

22. Table 1: Include at the end of the legend: “Days between recaptures are shown as mean (range)”. Exclude “mean (range)” from the table. Also, standardize decimal digits.

**Answer:** Done.

23. Line 349: I suggest “Courtship and oviposition behaviors”

**Answer:** Done.

24. Figure 3: Some adjustments in the legend: “(A) Female approaching the resident male in vocal activity. The white arrow indicates the location of the resident male in the bromeliad leaf-tank. On the left, the satellite male was observing the interaction. (B) In another set of bromeliads, the same female was inspecting the bromeliad leaf-tank while the resident male was in the leaf-tank above (white arrow). (C) Pair in amplexus in the leaf-tank inspected by the female. (D) After egg laying was completed, resident male left the female and sat on the upper leaf-tank, where he began to emit a courtship call to attract the female again. (E) Pair in amplexus in the upper leaf-tank. Notice the spawn just placed in the anterior leaf-tank and the approach of the satellite male (white arrow). (F) Satellite male sitting on the newly deposited spawn (white arrow). The pair remained in amplexus in the leaf-tank above”.

**Answer:** Done.

25. Line 396: substitute “couple” for “pair”. In the same line, substitute “the couple were” for “the pair was”. Check throughout the text.
**Answer:** Done. We replaced the word "couple" for the word "pair" throughout the text.

26. Line 431: correct “Spawns were found”

**Answer:** Done.

26. Figures 5, 6: It would be nice if photos could be arranged in the same order they are mentioned in the text, or maybe change the order in the text? For instance, at the beginning of the tadpole description (line 483), it is possible to mention Fig 5A-D.
**Answer:** Accordingly, we mentioned Fig 5A-D at the beginning of the tadpole description (line 483). Regarding to Fig 6 we tried to arrange the photos in the same order they are called in the text. However, in the case of figures 5 and 6, this is difficult because in the description we always refer to a general aspect of the tadpole (Fig 5), but describing the details of characters (Fig 6) in the sequence. To solve this, we would have to change the description, making it more redundant, or merge the figures 5 and 6, which would be unfeasible due to the number of photos. We think that this problem can be minimized if the plates are positioned near each other in the final version.

27. Line 581: There is no Fig 8 anymore. To which figure do you refer here?
**Answer:** The Fig 8 has been incorporated into Fig 5, in this case the reference is Fig 5D. We corrected it in the text.

28. Table 3: “Acoustic parameters of the calls of *Bokermannohyla astartea*…”

**Answer:** Done.

**Discussion**

29. Lines 688-695: It is always nice to initiate the discussion calling attention for the main results. Thus, I suggest beginning with the discussion about the use of bromeliads as calling and breeding sites and the record of a bromeligenous species in the tribe Cophomantini for the first time. It is easy to be done by changing the position of the sentences.
**Answer:** Done. We moved this part of the text to the beginning of discussion.

30. Lines 685-686: Discussion on the reproductive period should be moved to the end of the paragraph. Moreover, it would be nice to add a sentence about the occurrence of the species in the Atlantic forest, where annual precipitation is high mainly in spring/summer, which may favor the prolonged breeding season of the species and the use of bromeliads to reproduce.
**Answer:** Done. We moved this part of the text to the end of the paragraph and added a sentence about the seasonality of the species as suggested.

31. Lines 686-687: As I mentioned in the results, sexual size dimorphism should be discussed in the second paragraph. Males and females exhibited differences in SVL. Which differences? Clearly state that females were larger and mention that this is common pattern for anurans (with some references), although other *Bokermannohyla* species do not present sexual size dimorphism.
**Answer:** Done. We moved this part of the text to the second paragraph, and improved the text with discussion about the differences in SVL between males and females of *B*. *astartea*.

32. Line 719: substitute “positioning themselves over” by “sitting on”
**Answer:** Done.

33. Lines 720-722: rewrite as follows: “…when opportunistic males try to fertilize oocytes released by the amplectant female, which were not fertilized by the resident male...”
**Answer:** Done. We rewrote the sentence as suggested.

34. Line 725: I suggest “Courtship and oviposition behaviors”
**Answer:** Done.

35. Line 732: substitute “conduct” by “conducting”
**Answer:** Done.

36. Line 737: include “possibly” before “because”
**Answer:** Done.

37. Line 741-742: Courtship precedes amplexus and oviposition, thus I suggest rewriting as: “We recorded the partitioning of spawns among different leaf-tanks by amplectant females of *B*. *astartea*.”
**Answer:** Done.

38. Line 748: Partitioning of spawns has also been recorded for the hylid *Dendropsophus haddadi* (Silva et al. 2019. Reproductive biology of *Dendropsophus haddadi* (Bastos and Pombal, 1994), a small treefrog of the Atlantic forest. Herpetology Notes 12: 319-325). Given that Hylidae is a speciose family, I suggest adding that spawn partitioning might be common, although poorly reported.
**Answer:** Done. We have included the information and the respective reference in the manuscript.

39. Line 754: Emphasize your findings about the reproductive mode by adding “novel for anurans” at the end of the sentence.

**Answer:** Done.

40. Line 773: The correct would be Table 2 (tadpole measurements).

**Answer:** Done. We corrected it in the text.

41. Line 774: reword “bromeliad leaf-tanks”.
**Answer:** Done. We corrected it in the text.

42. Lines 777-780: It is intriguing why only tadpoles up to stage 26 occur in bromeliads. Why not until stage 21 or 24? There is a paper by Leite and Eterovick (2010), where they describe the tadpole of *Bokermannohyla martinsi* and make interesting comparisons among *Bokermannohyla* tadpoles regarding their morphology and ecology. In this paper, the authors suggest that *Bokermannohyla* tadpoles have a long duration of stage 25, probably related with the fact that most species reproduce in permanent ponds/streams. Do you think that the long duration of stage 25 could explain why only tadpoles up to stage 26 were found in bromeliads? Or, alternatively, is it possible that tadpoles have a mechanism of stop developing and growing while inside the bromeliads? There are many studies describing plasticity in tadpole growth and development (depending on environmental conditions) and I think it would be interesting to raise some of these hypotheses.
**Answer:** This is an interesting topic. They probably occur in bromeliads up to stage 26, because after that they would reach a maximum size unfeasible for living in small space. We added in the results information about the development stage of the tadpoles when they hatch of the eggs in bromeliads (stages 23-24). Thus, our data shows that only the initial and small free-living stages 23-26 would occur in bromeliads. The development/growth patterns mentioned for *B*. *martinsi* also occur in some species of the *B*. *pseudopseudis* group (Cardoso 1983; Eterovick & Brandão 2001; Lugli & Haddad 2006b; Lins et al 2018) but were not observed in species of the *B*. *circumdata* group (e.g., Mogin & Carvalho-e-Silva, 2013, Pezzuti et al. 2015). In species of this group, the correlation between development and growth seems to be more concerted. As we did not present data of development and growth, nor observed these patterns in nature, we are unaware to discuss which patterns *B*. *astartea* presents. Anyway, as we did not find large tadpoles in stage 25, it is unfeasible that they retain this stage for a long time. Although it is an interesting topic, we think that discussing this topic (on development and growth) without presenting these data, would increase the size of the manuscript, without bringing new information.

43. Line 856: substitute “between” for “with”
**Answer:** Done.

44.Line 858: reword: “may also have”
**Answer:** Done.

45. Lines 860-874: As I mentioned in the introduction, there is a misinterpretation of the study by Silva et al. (2020) about reproductive modes and sexual size dimorphism (SSD), and no sexual selection mechanisms are involved. I would suggest removing this discussion on SSD. However, if you decide to keep, I suggest reorganizing this paragraph, as follows:
“… some traits of the reproductive biology of B. astartea agree with a recent complementary hypothesis that is linked to sexual selection [5]. For example, the territoriality of males related to the sets of bromeliads and hidden amplexus and spawning may decrease the risk of polyandry [37, 5] and can also reduce spawning damage by multiple male harassment [5]. Besides male-male competition, terrestrial reproductive modes have been suggested to influence sexual size dimorphism and fecundity in anurans [7]. The less pronounced sexual dimorphism in relation to the SVL between males and females of B. astartea may occur because amplectant females do not carry males to another site for spawning [7]. Still, this space limitation of bromeliad leaf-tanks may favor the reduction of female size since the pair has to fit in the small space to spawn (e.g., [37, 100]), possibly reducing spawning size in these microhabitats, and thus resulting in a less pronounced sexual size dimorphism [7]. These traits are postulated to be strongly related to the evolution and diversification of terrestrial/arboreal reproductive modes in frogs, and reinforces that other selective pressures can act beyond predation and competition [5, 7].”

**Answer:** Thank you for this correction in the text. We corrected the part that had been misinterpreted and decided to keep the mention of SSD in the discussion, considering your suggested text.

46. Line 875: include “anuran” before “species”
**Answer:** Done.

47. Line 879: include “the” before “Atlantic forest”
**Answer:** Done.

48. Line 881: I suggest “…that favor the occurrence of many types of humid microhabitats…”
**Answer:** Done.

49. Line 885: I suggest “…basic biology of Neotropical anurans…”

**Answer:** Done.
